# SLC16A7 Promotes Triglyceride Deposition by De Novo Lipogenesis in Chicken Muscle Tissue

**DOI:** 10.3390/biology11111547

**Published:** 2022-10-22

**Authors:** Yongli Wang, Lu Liu, Xiaojing Liu, Xiaodong Tan, Yuting Zhu, Na Luo, Guiping Zhao, Huanxian Cui, Jie Wen

**Affiliations:** 1 State Key Laboratory of Animal Nutrition, Key Laboratory of Animal (Poultry) Genetics Breeding and Reproduction, Ministry of Agriculture, Institute of Animal Sciences, Chinese Academy of Agricultural Sciences, Beijing 100193, China; 2 College of Animal Science and Technology, College of Veterinary Medicine of Zhejiang A&F University, Hangzhou 311300, China

**Keywords:** triglyceride, de novo lipogenesis, intramuscular fat, SLC16A7, myocytes

## Abstract

**Simple Summary:**

Triglyceride (TG) content was decisive to intramuscular fat (IMF) deposition in poultry muscle, and IMF affected meat quality in many ways. Therefore, we identified key candidate genes affecting TG traits by a genome-wide association study (GWAS) on TG content traits. The main findings show that SLC16A7 (solute carrier family 16 member 7) was a key candidate gene affecting TG content, affecting TG deposition in myocytes through de novo lipogenesis.

**Abstract:**

Triglyceride (TG) content in chicken muscle tissue signifies intramuscular fat (IMF) content, which is important for improving meat quality. However, the genetic basis of TG deposition in chicken is still unclear. Using 520 chickens from an artificially selected line with significantly increased IMF content and a control line, a genome-wide association study (GWAS) with TG content reports a region of 802 Kb located in chromosome 1. The XP-EHH and gene expression analysis together reveal that the solute carrier family 16 member A7 (SLC16A7) gene is the key candidate gene associated with TG content in chicken muscle tissue. Furthermore, the weighted gene co-expression network analysis (WGCNA) confirmed the regulatory effects of SLC16A7 on promoting TG deposition by de novo lipogenesis (DNL). Functional verification of SLC16A7 in vitro also supports this view, and reveals that this effect mainly occurs in myocytes. Our data highlight a potential IMF deposition pathway by DNL, induced by SLC16A7 in chicken myocytes. These findings will improve the understanding of IMF regulation in chicken and guide the formulation of breeding strategies for high-quality chicken.

## 1. Introduction

Chicken meat is the second-largest meat product marketed for human consumption worldwide due to its taste, tenderness, and juicy quality. With the use of continuous growth breeding and high-density feeding to obtain more meat, the quality of the meat decreases due to the resulting relatively higher water and lower fat content [1]. At present, improving meat quality is the focus of intensive scientific research. Intramuscular fat (IMF) content has an important role in various quality traits of meat [2,3,4,5]. For example, the quality of Kobe beef meat is known to be due to its higher amount of IMF marbling. Therefore, the elucidation of the IMF deposition molecular regulatory mechanism in chickens is a hot research topic.

IMF is a mixture of various lipids, mainly including triglycerides (TG), phospholipids (PLIP) and cholesterol (CHO), which have different formation pathways [6,7]. These mixtures are deposited in muscle tissue. Fatty acids (FAs), a form of energy conversion and a TG and PLIP structural component, play a regulatory role in animal biological activity [2,8,9]. Triglycerides are formed by the esterification of fatty acids and glycerol. FAs sources in animal tissues include de novo lipogenesis (DNL) and cellular uptake. FAs for cellular uptake are derived from blood lipids and lipoproteins catabolized by lipoprotein lipase (LPL) [10,11]. At the same time, intracellular DNL synthesizes carbon-16 FA (palmitic acid) using acetyl coenzyme A and malonyl coenzyme A (malonyl-CoA) as substrates [12,13]. DNL mainly occurs in the poultry’s liver [12,13,14,15], and the necessary FAs are provided by cellular uptake to other tissues.

TG content is a complex quantitative trait controlled by polygenes [16]. However, the genetic basis and the key genes controlling the TG content trait in chicken muscle tissue are still unclear. Our team artificially bred Jingxing yellow-feather (JXY) chickens with IMF content in breast muscle tissue as the main selected trait for the last 20 years [17], providing a useful experimental model for the regulatory mechanism of IMF deposition in chickens. Previous studies have shown that TG content in chicken muscle tissue can represent the IMF content and closely correlate with fatty acids from the de novo synthesis and cellular uptake [18]. In this study, the selected and the corresponding control JXY chickens lines were used to investigate the candidate genes related to TG deposition through a systematic research approach involving a genome-wide association study (GWAS), selection signature analysis, and gene expression analysis, and further verified the function of candidate genes through cell experiments.

## 2. Materials and Methods

### 2.1. Animals and Sample Collection

Five hundred twenty female JXY chickens (*n* = 520; including 256 in the selected line and 264 in the control line) were obtained from the Institute of Animal Science, Chinese Academy of Agricultural Sciences (Beijing, China). The two JXY chicken lines originated from the same base population of JXY100, with IMF in breast muscle tissue as the main selection trait since 2000. The selected line was selected for increased IMF, and the control line was randomly bred. In each generation, at least 3 male and 3 female chickens within a full-sib family were euthanized, IMF content was measured, and the IMF means within a family were used, as previously described [18]. All birds were raised in three-stair step cages (one bird per cage) under the same nutritional conditions. The feeding cycle lasted from 1–98 days old, and the chickens were fed in the same batch according to the chicken-feeding standard (NY/T33-2004). All animals had free access to water and food. The chicken house maintained appropriate environmental temperature, relative humidity, and light for routine epidemic prevention and immunization.

For genomic DNA (gDNA) extraction, the blood samples from 520 chickens were collected using anticoagulant for genomic DNA extraction. Subsequently, chickens were euthanized under carbon dioxide anesthesia by severing the carotid artery at 98 days of age. Then, breast muscle tissue was dissected and stored at −20 °C or −80 °C for subsequent analyses.

### 2.2. Trait Measurements

A 2.0-g sample of each breast muscle tissue of JXY chickens was homogenized, then the TG content was measured using commercially available kits (Beijing Deliman Biochemical Technology Co., Ltd., Beijing, China).

A 5.0-g breast muscle tissue sample of each of JXY chicken was freeze-dried and ground for FAs extraction. Then, the FA composition was determined by gas chromatography (GC) according to a method previously reported using an HP6890 gas chromatograph (Hewlett-Packard Agilent, Palo Alto, CA, USA) [19].

### 2.3. Whole-Genome Re-Sequencing

The gDNA was extracted from blood samples using the phenol-chloroform method. A total of 520 gDNA samples were used for whole-genome re-sequencing. The quality and quantity of the gDNA were determined using a NanoDrop spectrometer (Thermo Fisher Scientific Inc., Waltham, MA, USA) and agarose gel electrophoresis. Then, the paired-end libraries were generated for each eligible sample using a standardized procedure for Illumina MiSeq data quality control. The average insert size was 300–500 bp. After quality control, all qualified libraries were sequenced with PE150 (150 bp double-ended) on a Novaseq 6000 sequencing platform (Illumina, San Diego, CA, USA) by Beijing CapitalBio Technology Co., Ltd. (Beijing, China) with at least 10 Gb of raw data per individual. The low-quality reads were filtered using the fastp software (https://github.com/OpenGene/fastp) (accessed on 10 July 2021) (parameters: -q 30; -u 30; -l 75), and the clean reads were aligned to the reference genome (Gallus_gallus-6.0) (GCA_000002315.5) using BWA MEM algorithm (version 0.7.10) with default parameters. Then, the duplicate reads were removed by SAMtools (version 1.9) and Picard MarkDuplicates (http://broadinstitute.github.io/picard) (accessed on 10 July 2021), and the mapped reads were converted into BAM files using SAMtools for the subsequent analysis [20,21].

### 2.4. Variant Discovery and Annotation

The base quality score recalibration (BQSR) was performed using exclusively GATK36 (version 3.5) and the new BAM files were output by rectifying the base quality in the original BAM files using ApplyBQSR according to a recalibration table, generated by the comparison of the original BAM files using BaseRecalibrator. The single-sample gvcf file was obtained by the HaplotypeCaller detection of new BAM files, and merged into a whole gvcf file using the CombineGVCFs software for population analysis. Next, the joint genotyping of multiple samples was performed using the GenotypeGVCFs software to obtain accurate genotypes. Then, the single nucleotide polymorphisms (SNPs) were filtered based on SelectVariants and VariantFiltration. Ultimately, the identified SNPs were annotated by the ANNOVAR software based on the gene annotation of the reference genome in Ensemble [22].

### 2.5. Genome-Wide Association Study (GWAS) Analysis

First, a quality control analysis of the genotype and phenotype data was performed. The phenotype batch effect was considered and individuals with missing or abnormal phenotypes were excluded. Genotype data were filtered with the standard as follows: (1) retaining all SNPs on chromosome 1–33; (2) the detection rate of all SNPs was greater than 90%; (3) minor allele frequency (MAF) was greater than or equal to 0.05; (4) the detection rate of an individual was greater than 80%. The filtered SNPs were subjected to the site filling treatment using the Beagle 5.0 software, for use in subsequent analysis [23].

An efficient mixed linear model (MLM) was used for the association analysis using the genome-wide efficient mixed-model association (GEMMA) software. The calculation model was Y = Xα+ Zβ +Wμ+ e, where Y is the phenotypic value, X is the indicator matrix of fixed effect, α is the fixed effect (batch effect and population structure PCA), Z is the indicator matrix of SNP, β is the SNP effect, W is the indicator matrix for random effects, μ is the random effects, and e is the residual item) [24]. The genetic relationship matrix was established on the SNPs after quality control, and the SNP significance was tested by the Wald test method. The significance threshold was obtained by the Bonferroni multiple-testing correction. The genomic significance level threshold was set as 0.05/effective SNP number. The threshold of chromosome significance level was set as 1/effective SNPs number.

### 2.6. Signature Selection Analysis

In this study, the signature of selection analysis was performed by the cross-population extended haplotype homozygosity (XP-EHH), based on the linkage unbalance principle. The extended strong LD region in the selected and control lines were analyzed using Selscan software, and the suggested −2/2 was used as the significant threshold line. The whole genome was scanned with a sliding window (10 Kb window size and 2 Kb step size) to reduce the false discovery rate.

### 2.7. RNA Sequencing and Data Analysis

In this study, our previous RNA-sequencing data, which analyzed the gene expression profiling in breast muscle tissue at different developmental stages (E12, E17, D1, D7, D21, D56, D98, D140, D180) (*n* = 3, total 24 birds), were used to investigate the SLC16A7 expression profile [25]. The reported raw sequence data were deposited in the Genome Sequence Archive in BIG Data Center, Beijing Institute of Genomics (BIG), Chinese Academy of Sciences, under project number PRJCA001192 and accession number CRA001334, which are publicly accessible at http://bigd.big.ac.cn/gsa (accessed on 10 July 2021).

In addition, the breast tissue samples of 16 individual 520 JXY chickens with high- or low-TG content were used for RNA sequencing. Based on ultra-high-throughput sequencing analysis (HiSeq2500; Illumina, San Diego, CA, USA), gene expression profiling was performed by Berry Genomics (Beijing, China). Raw data were converted to FASTQ files using bcl2fastq (Illumina). Clean reads were generated by removing reads with adapter and low-quality sequences and mapped to the reference chicken genome and genes (*Gallus gallus*, Galgal 6.0) using TopHat 1.3.2 (https://ccb.jhu.edu/software/tophat) (accessed on 10 July 2021). Gene expression levels were calculated using the method of Reads Per Kilobase of transcript per Million (RPKM). Differentially expressed genes (DEGs) between the two lines were analyzed using the edgeR R package. DEGs were screened by the following criteria: fold change ≥ 1.5, with *p* < 0.05. The KEGG (Kyoto Encyclopedia of Genes and Genomes) pathway enrichment analysis using Kobas3.0 [26]. *p* < 0.05 was considered to be indicative of statistical significance.

### 2.8. Weighted Gene Co-Expression Network Analysis (WGCNA)

Using the previous RNA-sequencing analysis data (Accession number CRA001908; http://bigd.big.ac.cn/gsa) (accessed on 10 July 2021), the 16 individuals from the JXY chickens, the association between gene sets, and phenotypes (FA composition, IMF, and TG) were respectively performed by the weighted gene co-expression network analysis (WGCNA). This algorithm filters genes with the top 25% variance as input data. The WGCNA was performed using the “WGCNA” package in the R software with an adjacency matrix, a topological overlap matrix (TOM), and a calculation of the corresponding dissimilarity (1-TOM). Gene dendrogram construction and module identification were performed using a dynamic tree cut, and correlations between the module genes and phenotype were calculated [27]. Further analyses of the module containing the target candidate genes were performed for gene expression analyses.

### 2.9. Construction of Over-Expression Vector of SLC16A7

The amplification primers for the SLC16A7 gene sequence were as follows: Forward primer: 5′-ctctagactcgaggtgaattcATGCCACCAGCAATAGGAGC-3′, Reverse Primer: 5′-gtagtcggatcctttgaattcAATATTGGTTTCTCTTTCTGAAGGATC-3′. The target fragment was amplified from myocytes’ cDNA library by standard PCR technology and high-fidelity DNA polymerase. The target fragment was amplified from myocytes’ cDNA library by standard PCR and high-fidelity DNA polymerase. The target gene fragment was ligated to the pcDNA3.1 expression vector and transformed into DH5α cells. The transformants were cultured on LB solid medium containing ampicillin overnight. The colonies were identified by PCR, and the successfully constructed pcDNA3.1-SLC16A7 plasmid was verified by sequencing and comparison of positive clones.

### 2.10. Primary Cells Isolation, Culture and Transfection

The primary intramuscular preadipocytes and myocytes were isolated from the major breast tissue of 7-day old chickens using a method based on a previous report [28]. All cells were incubated in Dulbecco’s modified Eagle’s medium (DMEM)/F12 medium with 10% fetal bovine serum (FBS, Gibco, Grand Island, NY, USA) in a humidified atmosphere of 5% CO_2_ at 37 °C. After reaching 80% confluence, cells were passaged after detaching with 0.25% trypsin-EDTA (Grand Island Biological Company (Gibco), Grand Island, NY, USA). The intramuscular preadipocytes and myocytes at the second passage were plated in 6-well or 100-mm dishes and cultivated for 24 h. Some of the cells were collected for subsequent experiments.

The myocytes were inoculated into 6-well plates, and SLC16A7 overexpression experiments were performed when the density reached 60–70%. Using Lipofectamine 3000 Transfection Reagent (Invitrogen, Carlsbad, CA, USA), over-expression vector of SLC16A7 (pcDNA3.1-SLC16A7) was transfected, and pcDNA3.1 vector was used as a control group.

In addition, the myocytes were inoculated in 100-mm plates for 24 h; when the density was about 90%, the cells were washed twice with PBS and then replaced into DMEM medium (Gibco, Grand Island, NY, USA) containing glucose (8 mM), pyruvate (25 mM) and sodium lactate (15 mM), respectively. The normal culture was used as the control group, and other experimental conditions were the same. After 48 h, myocytes were collected and lysed in lysis buffer (Promega, Madison, WI, USA) for further phenotypic detection. myocytes total RNA was isolated using TRIzol reagent (Ambion, Carlsbad, CA, USA). SLC16A7 mRNA expression level was detected by real-time PCR. At the same time, the protein of myocytes was isolated, and the protein expression level was detected by Western blot analysis.

### 2.11. Quantitative Real-Time Polymerase Chain Reaction (qRT-PCR)

Total RNA was extracted from each breast tissue and cell sample using TRIzol reagent (Invitrogen, Carlsbad, CA, USA) according to the manufacturer’s instructions. The quantitative real-time polymerase chain reaction (qPCR) was performed using SYBR Green reagents on a 7500 Real-Time PCR System (Applied Biosystems, Foster City, CA, USA). The specific primers of the SLC16A7, ACACA, FASN genes are listed in Table S1. Gene expression was normalized using *18S RNA* by using the 2^−^^ΔΔ^^Ct^ method. Each sample was analyzed in triplicate with standard deviations of CT values not exceeding 0.5 on a within-run basis.

### 2.12. Western Blotting

The cells were lysed with RIPA buffer (Solarbio, Beijing, China) at 4 °C and the total protein was extracted. Protein was diluted in 6× Protein loading buffer (TransGen Biotech, China) and denatured at 95 °C. The protein samples were separated by 12.0% SDS-PAGE and transferred to 0.22 μm PVDF membrane at 350 mA for 60 min. The membrane was blocked with 5% milk dissolved in TBST.  Membranes were incubated with SLC16A7 antibody (1:1000, Anti-SLC16A7 rabbit polyclonal antibody, Sangon Biotech, China) and ACTIN antibody (1:5000, Gene-Protein Link, China) for 60 min at room temperature. After washing with TBST, the membrane was incubated with secondary anti-rabbit IgG (1:10,000; Abmart, China) for 60 min. The results were analyzed using the Tanon-410 automated gel imaging system (Tanon, China). Image J software was used for quantification and data analysis.

### 2.13. ^13^C Isotope Tracing Technology

The cells were inoculated into 100-mm plates and all cells were kept at 37 °C in a humidified atmosphere with 5% CO_2_ for 24 h. The cells were washed twice with PBS to remove excess glucose from the medium, then incubated in a medium containing [U-^13^C]-glucose and phenol red -free DMEM for 96 h. Next, 2 mL mixture liquid (water: methanol: chloroform volume ratio = 1:1:2) were added. Cells were lysed by 5 cycles of 1 min ultra-sonication and 1 min rest intervals in an ice-water bath. The extraction solution was centrifuged at 3500× *g* and 4 °C for 10 min. The chloroform layer was removed, dried with nitrogen, then dissolved in 0.5 mL of 0.5mM potassium hydroxide in 75% ethanol in a water bath at 80 °C for 1 h, and allowed to cool. After samples were cooled, 600 μL hexane were added and samples were centrifuged at 4 °C for 10 min. The supernatant was evaporated to dryness under nitrogen, and derivatized in 20 μL 1-Hydroxybenzotriazole (HoBt), 40 μL cholamine, and 20 μL 2-(7-Azabenzotriazol-1-yl)-N,N,N′,N′-tetramethyluronium hexafluorophosphate (HATU). Equal volume samples from the prepared samples were mixed as quality control samples. UHPLC-HRMS analysis was performed, and the data collection of the metabolite spectrum was shown in the literature [29]. The analytical software Xcalibur (version 4.0.27.19) was used to view the spectrum and integrate the target metabolites. At the same time, a manual inspection was performed to ensure the accuracy of the integrated area of each target, export the data, and obtain the original integrated area. Reference method, natural isotope correction of original data [30].

### 2.14. Enzyme-Linked Immunosorbent Assay (ELISA)

The malonyl-CoA content in transfected pcDNA3.1-SLC16A7 cells and the control cells were measured using a chicken-specific ELISA kit (Enzyme-linked Biotechnology Co., Ltd., Shanghai, China). Cell samples were homogenized using a whirlpool oscillation at room temperature and centrifuged at 1000× *g*, at 4 °C for 20 min to separate the debris and the pellet. The supernatant was frozen immediately at −80 °C until assayed. The assay was conducted following manufacturer protocols and suggested dilutions to optimize accuracy.

### 2.15. Statistical Analyses

The significance of differences between groups was tested by the Student t-test using the SPSS Version 22.0 (IBM Cop., Armonk, NY, USA). Confidence limits were set at 95%, and *p* < 0.05 (*) or *p* < 0.01 (**) was considered significant. Data are presented as the mean ± standard deviation (SD).

## 3. Results

### 3.1. Genome-Wide Association Study (GWAS) of TG Content in the JXY Chicken Breast Muscle Tissue

**Figure 1:**
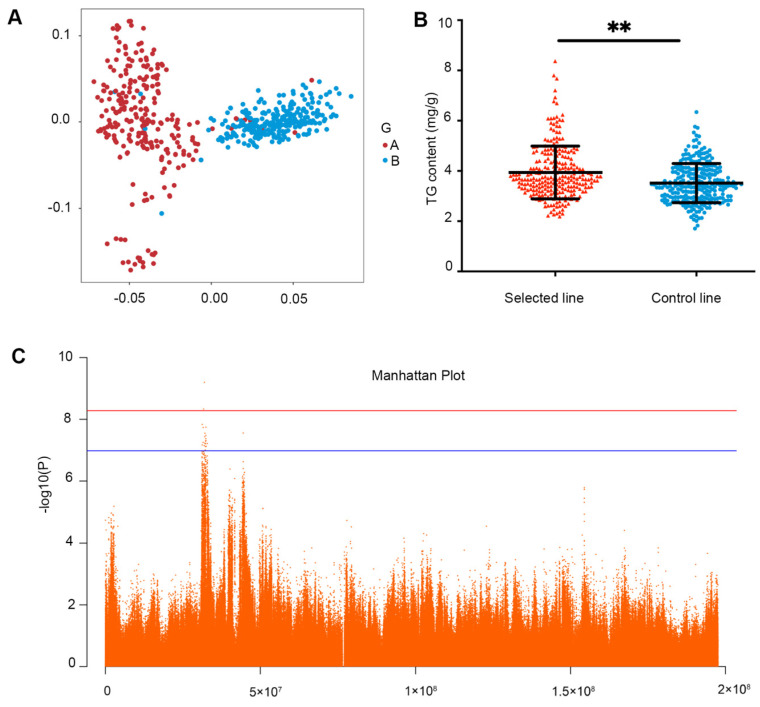
The GWAS result of TG content in the breast muscle tissue of Jingxing-yellow (JXY) chickens. (**A**) The principal component analysis (PCA) of the population structure of the selected and the control lines (A stands for the selected line, B stands for the control line). (**B**) TG content of the selected line and the control line (**, *p* < 0.01). (**C**) Genome-wide association analysis of significant regions for TG traits. The red and blue lines indicate the Bonferroni-corrected thresholds of genome-wide and suggestive significances, respectively.

Using 520 female JXY chickens from the selected (*n* = 256) and control (*n* = 264) lines at the 16th generation, we performed whole-genome re-sequencing with a mean coverage depth of 10×. In total, 17,915,382 SNPs were examined in the entire genome (accession numbers CRA002643 and CRA002650, https://bigd.big.ac.cn/gsa) (accessed on 10 July 2021) and 9,614,883 SNPs were used for GWAS analysis of TG traits after a genotype-imputation treatment. The genetic variants were demonstrated according to the genome-wide significance line 0.05/9,614,883 (−log_10_ *p* = 8.28) and suggestive line 1/9,614,883 (−log_10_ *p* = 6.98). Additionally, the principal component analysis (PCA) revealed that the selected and control lines were divided into clusters with genetic variation (Figure 1A).

As the main constituent of IMF, the TG content was significantly higher in the breast muscle tissue of the selected line than in the control line (Figure 1B). After the effectiveness analysis of the phenotype data, 516 chickens with normal distribution were selected for GWAS (Table S2, Figure S1). The GWAS identified SNPs associated with TG content in the JXY chickens’ breast tissue, a 1.657 Mb region (chr1: 31,251,236-32,908,662) was screened in chromosome 1, including 24 SNPs with a significant *p*-value (Figure 1C and Figure S3, Table S3). These SNPs could explain 7.37% of the genetic variation of this trait.

### 3.2. Identification of Candidate Genes Associated with TG Content in Breast Tissue

**Figure 2:**
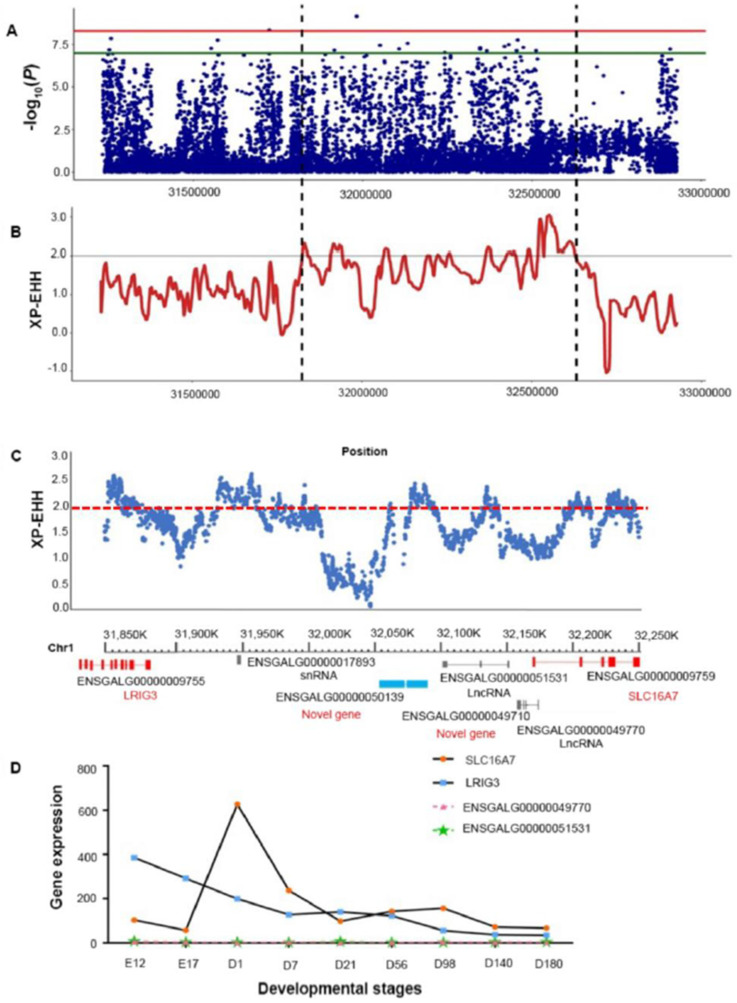
Gene-mapping of important candidate genes with regulatory effect on TG deposition in breast muscle tissue. (**A**) The region with the significant selection signature was the same as the region of the GWAS SNPs associated with TG content. (**B**) Minimize selection signature distribution within the candidate region. (**C**) The selection signature distribution (XP-EHH) of all sites within the candidate region is related to the gene. (**D**) Expression profile of breast muscle tissue of JXY chicken at different ages in the fourteenth generation (E stands for embryonic stage, D stands for postnatal age.

We also performed a selection signature analysis using the XP-EHH method. We found a significant selection signature in the same region containing the GWAS-identified SNPs associated with TG content (Figure 2A). Further, we minimized the associated region using the selection signature in the local XP-EHH analysis and highlighted the common region (chr1: 31,826,000–32,628,000) (Figure 2B). After gene annotation in the Ensemble database, and after eliminating the barren area of protein-encoding genes, the found the region in chr1: 31,826,000–32,250,000 contains four protein-encoding genes, including two known genes (SLC16A7 and LRIG3) and two novel genes (ENSGALG00000050139 and ENSGALG00000049710), as shown in Figure 2C.

Following that, we investigated the developmental expression profiles of SLC16A7, LRIG3, and the two novel genes (ENSGALG00000050139 and ENSGALG00000049710) in the breast tissue of JXY chickens using our previous RNA-sequencing data. The SLC16A7 and LRIG3 expression levels changed during development. In contrast, the two novel genes were not expressed (Figure 2D). Therefore, SLC16A7 and LRIG3 were considered the candidate genes associated with TG content in the breast tissue of JXY chickens. Further validations of the expression and effect of these genes were performed.

### 3.3. SLC16A7 Is an Important Gene Related to TG Biosynthesis in Muscle Tissue of Chicken

**Figure 3:**
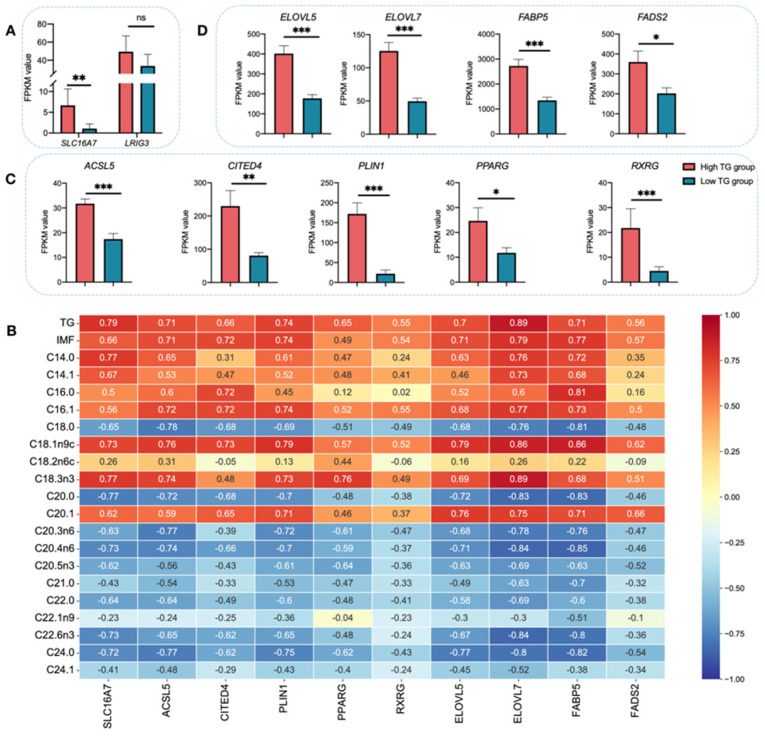
Association of SLC16A7 with the TG and poly fatty acid contents. (**A**) The expression of genes related to SLC16A7 and LRIG3 (** *p* < 0.01). (**B**) Relationship between lipid metabolism-related genes and TG, IMF and fatty acid traits in breast muscle tissue (MEbisque4 module). (**C**) The expression of genes related to TG esterification and adipocyte differentiation (* *p* < 0.05, ** *p* < 0.01, *** *p* < 0.001). (**D**) The expression of genes related to FA metabolism. (* *p* < 0.05, *** *p* < 0.001).

The previous transcriptome data (Table S4, accession number CRA001908, https://bigd.big.ac.cn/gsa) (accessed on 10 July 2021) of 16 JXY chicken breast muscle tissue from the selected (high-TG content, *n* = 8) and control lines (low-TG content, *n* = 8), with significant differences in the TG content, were used to find differentially expressed genes (DEGs). We compared the expressions of the SLC16A7 and LRIG3 genes using data from 16 chickens to further identify candidate genes affecting TG traits. Notably, the SLC16A7 mRNA level was significantly higher in selected chickens than in control chickens, but the LRIG3 mRNA was not different between these two groups (Figure 3A). WGCNA were performed using the transcriptome data (Table S4) of 16 JXY chicken breast muscle tissue with 27 traits (including TG and FAs) (Table S5). We identified 22 modules after merging the modules (Height value < 0.25) and calculated the correlation of these gene modules with phenotypic traits (Figure S2). Additionally, we screened the significant modules by correlation analysis between the modules and traits, including the MEbisque4, etc., mainly involved in TG, and in C14:0, C14:1, C16:0, C16:1, and C18:1n9c traits (Figure 3B). Additionally, SLC16A7 was enriched in the MEbisque4 module, containing 2117 genes (Table S6). The results showed a significant correlation between SLC16A7 expression and TG content (r = 0.79, P = 7 × 10^−3^), C14:0 (r = 0.77, P = 7 × 10^−4^), C14:1 (r = 0.67, P = 7 × 10^−3^), C16:0 (r = 0.50, P = 5 × 10^−2^), (r = 0.56, P = 3 × 10^−2^), and C18:1n9c (r = 0.73, P = 2 × 10^−3^) (Figure 3B) in breast muscle tissue.

As shown in Figure 3B, we also identified nine other representative genes related to lipid metabolism in the MEbisque4 module co-expressed with SLC16A7, which also significantly correlate with the content of TG, C14:0, C14:1, C16:0, C16:1, or C18:1n9c, mainly involved in TG esterification or adipocyte differentiation (ACSL5, CITED4, PLIN1, PPARG, and RXRG), and FA metabolism (ELOVL5, ELOVL7, FABP5, and FADS2). The mRNA levels were also significantly higher in the high-TG group than in the low-TG group (Figure 3C,D).

### 3.4. Effect of SLC16A7 on De Novo Lipogenesis

**Figure 4:**
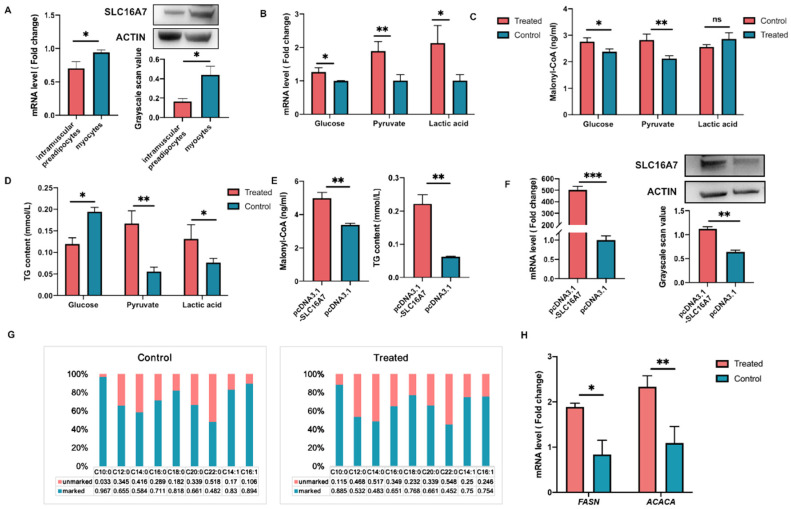
In vivo regulatory effect of SLC16A7 on TG synthesis via de novo lipogenesis. (**A**) The expression of the SLC16A7 gene in intramuscular preadipocytes and myocytes, mRNA expression level (left) and protein expression level (right). Error bars represent SEM (* *p* < 0.05). (**B**) SLC16A7 mRNA Expression level (* *p* < 0.05, ** *p* < 0.01). (**C**) The content of malonyl-CoA after exogenous addition of Glucose, pyruvate and sodium lactate were added externally for 48 h (* *p* < 0.05, ** *p* < 0.01, ns: not significant). (**D**) The content of TG after exogenous addition of Glucose, pyruvate and sodium lactate were added externally for 48 h (* *p* < 0.05, ** *p* < 0.01). (**E**–**H**) The high transfection efficiency of the overexpression vector of SLC16A7 gene, (**E**) the contents of TG and malonyl-CoA in myocytes (** *p* < 0.01), (**F**) The expression of the SLC16A7 mRNA (** *p* < 0.01, *** *p* < 0.001), (**G**) Relative proportions of ^13^C isotope-labeled and unlabeled fatty acids in myocytes, (**H**) The genes expression changes related to FA biosynthesis and lipid deposition after transfection for 48 h in myocytes (* *p* < 0.05, ** *p* < 0.01).

First, to identify the specific SLC16A7 expressing cell types, we analyzed the SLC16A7 mRNA expression in primary intramuscular preadipocytes and myocytes isolated from the chicken breast tissue. SLC16A7 is expressed in both intramuscular preadipocytes and myocytes, with higher expression levels in myocytes than in intramuscular preadipocytes (Figure 4A). Further, de novo lipogenesis (DNL), was stimulated with glucose, pyruvate, or sodium lactate in myocytes; with induction, the treated groups showed significantly higher SLC16A7 mRNA levels than the control groups (Figure 4B). More importantly, the exogenous addition of pyruvate promoted malonyl-CoA and TG contents also significantly increased (Figure 4C,D).

Further, we transfected an over-expression vector carrying the SLC16A7 gene into myocytes, with [^13^C_6_]-glucose replacing common glucose for 96 h. The malonyl-CoA and TG contents significantly increased in the treated myocytes compared with those in the control cells (Figure 4E), concomitant with SLC16A7 expression levels changes (Figure 4F). Additionally, the isotope tracer technique result showed that the proportion of the fatty acids in the de novo synthesis significantly increased in the treated myocytes compared with the control cells (Figure 4G). Moreover, over-expression of SLC16A7 in myocytes for 48 h induced changes in the expression levels of some representative genes related to FA biosynthesis (FASN, ACACA). These results revealed that the FASN and ACACA expressions were up-regulated (Figure 4H).

## 4. Discussion

IMF, the lipid mixture in the muscle tissue [6], is a complex quantitative trait with medium-low heritability [31,32]. It was confirmed that TG is a representative index of IMF in chicken, and its content determines the IMF content [33]. Therefore, the TG deposition regulation is critical to elucidating the genetic mechanism of IMF deposition in chickens. A good experimental model is critical to the success of scientific research [34]. Our team successfully bred a selected strain of JXY chickens with a consistent increase of IMF content in breast muscle tissue. Using 520 JXY chickens from the 16th generation of the selected line and control line, we identify SLC16A7 as the key gene on TG deposition in the muscle tissue of chickens.

Artificial selection can improve the performance of a target trait by changing the genome-wide genetic basis [35,36,37]. GWAS analysis has incomparable advantages in the systematic screening of the genetic variations of important quantitative traits, such as the body weight of duck and flavor quality of tomatoes [38,39,40]. To explore the genome-wide regulation of IMF deposition in the artificial selection of JXY chickens, we used a joint strategy of GWAS and selection signature analysis to identify the primarily associated genes. Accordingly, we found a GWAS locus with 24 significant SNPs associated with TG content in chromosome 1. After the selection signature analysis, we precisely located the region in the chr1:31,8260,000–32,250,000, including the protein-encoding SLC16A7, LRIG3, and two novel genes. GWAS and transcriptome data analysis combined strategy is a breakthrough in identifying functional genes associated with traits [41]. Additionally, the developmental expression profile analysis data of these four genes by RNA-sequencing support that the SLC16A7 and LRIG3 are the candidate genes for controlling the TG deposition process in chicken breast muscle tissue.

Notably, the SLC16A7 mRNA level is significantly higher in individuals with high-TG content than those with low-TG content. However, the LRIG3 mRNA level is not different between the two groups, suggesting SLC16A7 may have a more important role in TG deposition compared to LRIG3. Furthermore, a WGCNA revealed that the SLC16A7 expression level synchronously has significant correlations with TG contents and multiple FAs (C14:0, C16:0, C14:1, C16:1, and C18:1n9c) in breast muscle tissue. DNL mainly occurs in the liver of poultry [42], so the relationship of SLC16A7 expression with C14:0 and C16:0 contents, key intermediate or end products of DNL [43], suggested that the FA biosynthesis is involved in TG deposition in chicken muscle tissue. Genetically, SLC16A7 co-expressed with ACSL5, ELOVL5, ELOVL7, FABP5, FADS2, PLIN1, PPARG, and RXRG, involved in FA biosynthesis, elongation, desaturation, transport, and esterification [44,45,46,47,48,49]. Thus, SLC16A7 was considered a key gene, which might promote the TG deposition with DNL in chicken muscle tissue.

The physiological function of SLC16A7 in regulating TG deposition was confirmed by in vitro experiments. Firstly, we explored the SLC16A7 expression localization in muscle tissue cells. Myocytes and intramuscular preadipocytes are the main cell types in chicken breast muscle tissue [50], and SLC16A7 is mainly expressed in myocytes. SLC16A7 is known to play an important role in transporting pyruvate and lactate into the cell [51,52]. Additionally, the intracellular pyruvate and lactate can be involved in DNL to accelerate TG deposition by FA biosynthesis. Accordingly, the SLC16A7 effects on FA metabolism were also considered with SLC25A1, a similar transporter protein [53,54,55]. We found that the TG content was significantly increased after the over-expression of SLC16A7. SLC16A7 was significantly up-regulated after adding the inducer pyruvate, TG content increase, accompanied by the significant increase of ACACA and FASN mRNA levels, malonyl-CoA (as the initiation products of de novo synthesis of fatty acids), and the fatty acid carrying [U-^13^C] glucose by all related to the FAs de novo synthesis. These results supported the conclusion that SLC16A7 promotes TG deposition, and confirmed the important contribution of the DNL to TG deposition in chicken muscle tissue. Additionally, it indicated that myocytes were considered a considerable contributor to the IMF in chicken..

**Figure 5:**
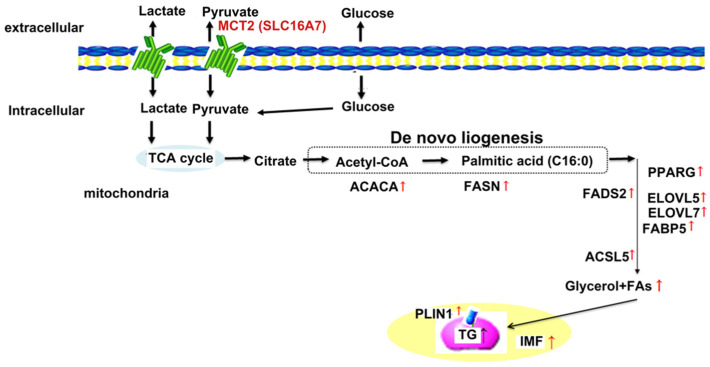
Potential metabolic pathways affecting TG synthesis of breast muscle in chicken. The black solid arrows indicate reported regulatory relationships.

TG content is a complex quantitative trait controlled by polygenes. The present study’s objective was to identify the key gene regulating TG deposition in chicken muscle tissue. Considering all of the above results, we propose a potential metabolic pathway by which SLC16A7 exerts its regulatory effect on TG deposition in chicken muscle tissue (Figure 5). These findings establish the groundwork and provide new clues for deciphering the molecular mechanisms underlying TG and IMF deposition in the muscle tissue of poultry. In vitro validation of SLC16A7 was performed, but no in vivo experiments were performed due to the limitations of gene-editing technology in chickens. Additionally, further studies on SLC16A7 in regulating TG deposition in the muscle tissue will be required to complement the molecular mechanisms. Increasing the IMF content is an important way to improve meat quality [2,3,4,5], as can be seen in Kobe beef, a classic, high-quality meat known for its marbling, tenderness, and taste. Our findings will provide new ideas and directions for breeding and nutritional regulation strategies for high-quality chicken.

## 5. Conclusions

In summary, our study identifies a regulatory pathway of triglyceride deposition in the muscle tissue of chickens. Additionally, our findings elucidate the regulatory effect of SLC16A7 on the increase of TG deposition with de novo lipogenesis, mainly in the myocytes of chicken muscle tissue. Our research improves the understanding of the IMF regulatory mechanism in chicken muscle tissue. Thus, it can help guide breeding and nutritional regulation strategies for high-quality chicken.

## Data Availability

The whole genome resequencing data and raw RNA-sequencing data in this paper have been deposited in the https://bigd.big.ac.cn/gsa/ (accession data code CRA002643, CRA002650 and CRA001908, accessed on 10 July 2021). Details of individuals with whole genome resequencing in this study provided in Supplementary Materials Table S7.

## Supplementary Materials

The following supporting information can be downloaded at: https://www.mdpi.com/article/10.3390/biology11111547/s1, Figure S1. The normal distribution of the population structure of the selected and the control lines (A stands for selected line, B stands for control line). Figure S2. Relationships between modules and phenotypic traits in pectoral muscle tissue. Figure S3. Manhattans and Q-Q plots of GWAS for TG content based on WGR data. Table S1. The specific primers for PCR in this study. Table S2. The phenotype data of triglycerides content in 516 chickens. Table S3. Annotation results of 24 SNPs with significant *p* values. Table S4. The transcriptome data of 16 JXY chicken breast muscle tissue from selected and control lines. Table S5. The traits of 16 JXY chicken breast muscle tissue with high- or low-TG content. Table S6. Gene enrichment results of the module where SLC16A7 is located (bisque4 module). Table S7. Detailed information statistics of 520 individuals.

## Abbreviations

IMF	intramuscular fat
TG	Triglyceride
GWAS	genome-wide association study
WGCNA	the weighted gene co-expression network analysis
PLIP	phospholipids
CHO	cholesterol
FAs	Fatty acids
SLC16A7	solute carrier family 16 member 7
LRIG3	leucine rich repeats and immunoglobulin like domains 3
ACACA	acetyl-CoA carboxylase alpha
FASN	fatty acid synthase
ACSL5	acyl-CoA synthetase long-chain family member 5
CITED4	Cbp/p300 interacting transactivator with Glu/Asp rich carboxy-terminal domain 4
PLIN1	perilipin 1
PPARG	peroxisome proliferator-activated receptor gamma
RXRG	retinoid X receptor gamma
ELOVL5	ELOVL fatty acid elongase 5
ELOVL7	ELOVL fatty acid elongase 7
FABP5	fatty acid binding protein 5
FADS2	fatty acid desaturase 2
SLC25A1	solute carrier family 25 member 1
DNL	de novo lipogenesis
GEMMA	genome-wide efficient mixed-model association
XP-EHH	the cross-population extended haplotype homozygosity
HoBt	1-Hydroxybenzotriazole
HATU	2-(7-Azabenzotriazol-1-yl)—N,N,N′,N′-tetramethyluronium hexafluorophosphate
