# Supplementary figures and images for: SLC16A7 Promotes Triglyceride Deposition by De Novo Lipogenesis in Chicken Muscle Tissue
Source: Biology (Basel). 2022 Oct 22;11(11):1547. doi: 10.3390/biology11111547 (PMC9687483; doi:10.3390/biology11111547)

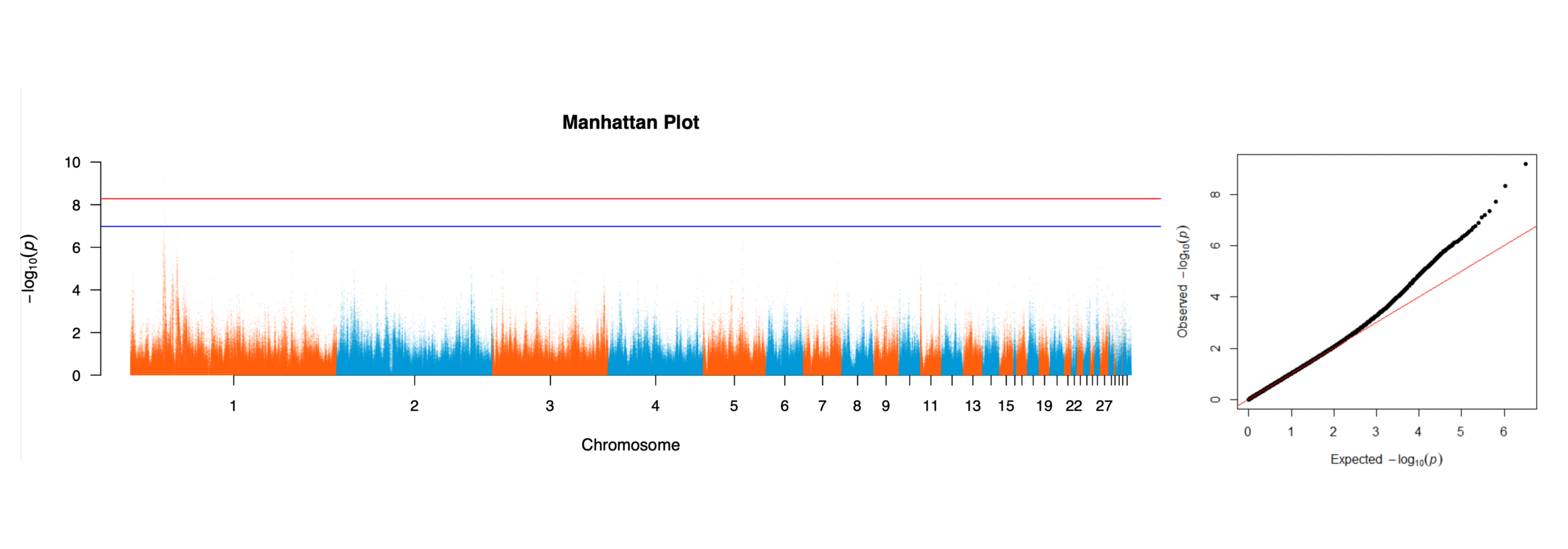

Supplement: Supplementary file 1 [file biology-11-01547-s001.zip › Figure S3.tif]
